# Consumer acceptance and preference for brown rice—A mixed‐method qualitative study from Nepal

**DOI:** 10.1002/fsn3.2803

**Published:** 2022-03-04

**Authors:** Pratiksha Gyawali, Dipesh Tamrakar, Abha Shrestha, Himal Shrestha, Sanju Karmacharya, Sanju Bhattarai, Niroj Bhandari, Vasanti Malik, Josiemer Mattei, Donna Spiegelman, Archana Shrestha

**Affiliations:** ^1^ Department of Biochemistry Dhulikhel Hospital – Kathmandu University Hospital Dhulikhel Nepal; ^2^ Department of Community Medicine Dhulikhel Hospital – Kathmandu University School of Medical Sciences Dhulikhel Nepal; ^3^ Department of Community Programs Dhulikhel Hospital Dhulikhel Nepal; ^4^ 2080 Department of Physiology, Anatomy and Microbiology La Trobe University Bundoora Victoria Australia; ^5^ Department of Nutrition Kathmandu University Hospital Dhulikhel Nepal; ^6^ 50296 Kathmandu University School of Medical Sciences Dhulikhel Nepal; ^7^ 1857 Department of Nutritional Sciences University of Toronto Toronto Ontario Canada; ^8^ Department of Nutrition Harvard TH Chan School of Public Health Boston Massachusetts USA; ^9^ Department of Biostatistics Yale School of Public Health New Haven Connecticut USA; ^10^ Center of Methods for Implementation and Prevention Science Yale School of Public Health New Haven Connecticut USA; ^11^ 50296 Department of Public Health Kathmandu University School of Medical Sciences Dhulikhel Nepal; ^12^ Department of Chronic Disease Epidemiology Yale School of Public Health New Haven USA; ^13^ Institute of Implementation Science and Health Kathmandu Nepal

**Keywords:** brown rice, perception, white Rice

## Abstract

**Background:**

Brown rice consumption reduces the risk of diabetes. The prevalence of diabetes is increasing in Nepal; however, dietary preference remains for white rice. This study aimed to understand the perception, enablers, barriers, and facilitators of acceptance brown rice at a worksite cafeteria.

**Methods:**

We conducted a mixed‐method qualitative research among 42 employees of a hospital in central Nepal. The participants tasted and rated the qualities of five different combinations of brown and white rice on a hedonic scale. We conducted eight focus group discussions (FGDs)—four before and four after tasting rice combinations. FGDs were recorded, transcribed, and coded verbatim and analyzed manually using inductive–deductive thematic method.

**Results:**

Before tasting, the participants perceived brown rice as poor in quality. After tasting, the participants found that brown rice had better quality and were willing to switch gradually starting with a 25B ratio. Eighty‐three percent of participants liked a combination of 25B. Major barriers were poor perception of its quality, tradition, unavailability, lack of awareness of health benefits, and high price. Major facilitators were availability, self and family awareness about the health benefits, knowledge, the brown rice cooking process, serving with side dishes, prior tasting, and gradual substitution of brown rice.

**Conclusion:**

We found that brown rice should be promoted stepwise, first as a mixture with white rice and gradually increasing the proportion of brown rice. Brown rice acceptance can be increased by improved knowledge of its nutrition and health benefits, increasing availability, and affordability.

## Graphical abstract

Brown rice is poorly perceived among Nepali consumers. The promotion of brown rice can be achieved in worksite cafeterias by gradually substituting with white rice, initially in lower proportion as a mixture of brown and white rice. Consumers are willing to switch to brown rice if they understand its health benefits and nutritive properties. Brown rice availability at a reduced cost will further enhance its acceptance.

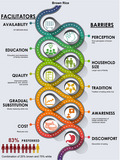

## 1 INTRODUCTION

There are 463 million people living with diabetes worldwide; these figures are expected to increase to 700 million by 2045 (Saeedi et al., 2019). In southeast Asia alone, 88 million people are living with diabetes and is projected to reach 153 million by 2045 (Saeedi et al., 2019). South Asian phenotypes with lower body mass index (BMI) and high body fat especially abdominal are known to have an increased predisposition for type 2 diabetes (Hills et al., 2018a). Furthermore, South Asians tend to develop diabetes at younger ages compared to the Caucasians (Mukhopadhyay et al., 2006). The decline in glycemic control over time is much more rapid among South Asians compared to Europeans (Mukhopadhyay et al., 2006). Progression from prediabetes to diabetes is also known to be more rapid among South Asians (Unnikrishnan et al., 2018). In Nepal, about 17% are overweight, 3% obese, and 9% of the population is estimated to have diabetes (Hills et al., 2018b).

The increase in diabetes prevalence in low‐ and middle‐income countries has been attributed to the nutrition transitions, especially shift in dietary consumption toward highly refined carbohydrates (Popkin, 2015). The typical South Asian meal is high in carbohydrate content (Misra et al., 2009). In the traditional diet, carbohydrates were typically derived from “under‐milled” grains such as hand‐pounded rice (Shobana et al., 2011). Currently, hand‐pounded rice has been replaced by polished white rice (refined grain) due to modern milling technologies to increase rice yield (Shobana et al., 2011). The degree of milling has an effect on the nutritional properties of brown rice and hence the glycemic index (GI) (Saleh et al., 2019). Moreover, GI of brown rice is lower as compared to the white rice (Mohan et al., 2014; Panlasigui & Thompson, 2006; Wordu & Banigo, 2013).

Habitual consumption of white rice has been associated with a higher risk of type 2 diabetes mellitus and metabolic syndrome among urban adult Indians, Chinese, and Caucasians (Radhika et al., 2009). Several studies have documented the beneficial effects of consuming whole grains to reduce postprandial blood glucose levels (Malik et al., 2019; Panlasigui & Thompson, 2006) and improve lipid profiles (Malik et al., 2019). Consumption of brown rice has been inversely associated with the risk of type 2 diabetes. Greater than 2 servings per week of brown rice compared to <1 serving per month was associated with a lower risk of diabetes, whereas greater than 5 servings of white rice compared to <1 serving per month was associated with a higher risk of diabetes (Sun et al., 2010). Several studies conducted in various populations have reported on the awareness and perception of brown rice. A focus group discussion conducted among Chinese adults found that awareness of the nutritional value of brown rice could potentially increase its acceptability over white rice (Zhang et al., 2010). Likewise, a study from India found that the major barriers to the acceptance of brown rice were lack of general awareness and knowledge of nutrition (Kumar et al., 2011).

However, no research to date has focused on the awareness and the acceptability of replacement of white rice for brown rice among Nepalese adults. Nepalese preferably consume white rice in the form of the traditional Nepali lunch set consisting of white rice, pulses, vegetables, pudding, and fried rice on a regular basis (Tamrakar et al., 2020). There is a need to understand the perceptions of brown rice, before we properly introduce brown rice in any setting. Therefore, this study seeks to understand how adults perceive brown rice and what factors can promote their acceptance from refined white rice to brown rice in a worksite cafeteria. In this mixed‐method study, we explored qualitatively how brown rice is perceived by Nepalese adults and what barriers and enablers exist for brown rice consumption. We also evaluated quantitatively the preference for brown rice, white rice, or their combination.

## 2 METHODS

### 2.1 Study design

This is an exploratory mixed‐method qualitative study conducted among the employees of Dhulikhel Hospital‐Kathmandu University Hospital (DH‐KUH), as a part of a formative study for Nepal Pioneer Worksite Intervention Study (NPWIS). Objective of the NPWIS is to measure the effect of cafeteria and behavioral intervention on the reduction of markers of cardiometabolic risk in Nepal (Shrestha et al., 2019). The current study was conducted in three parts: a before‐tasting FGD was conducted first, followed by tasting and rating of five different combinations of brown and white rice using hedonic rating scale, and subsequently concluded with an after‐tasting FGD.

### 2.2 Participant recruitment and sampling strategy

We obtained a list of employees who participated in the NPWIS screening for hypertension and diabetes in 2016. Out of 816 employees who were screened, we purposely selected and contacted 50 potential participants stratified by the presence or absence of central obesity. The study purpose and expectations were explained. We recruited 42 participants after receiving a written informed consent and 8 lost interest in the study. The participants represented two major groups: (1) medical group (doctors, nurses, paramedics, and laboratory personnel) and (2) nonmedical group (administrative, maintenance, housekeeping staff). Ethical clearance was obtained from the institutional review committee of Kathmandu University School of Medical Sciences. The data were collected between January 2017 and May 2017.

### 2.3 Focus group discussions

We conducted a total of eight focus group discussions: four before (*n* = 42) and four after (*n* = 42) the brown rice tasting. We developed semistructured and open‐ended questions for the FGD guide. Two investigators (PG, AS) reviewed the contents and pretested the guide among 11 healthy volunteers who were also the employees of DH‐KUH and not part of the study.

#### 2.3.1 Before‐taste FGD

The purpose of the before‐tasting FGD was to develop an understanding of factors influencing brown rice consumption. The before taste guide covered five major themes: (1) perception about brown rice, (2) rice preference, (3) knowledge about health effects of brown rice, (4) barriers to brown rice acceptance, and (5) facilitators of brown rice acceptance. A moderator (PG, DT, or AS) asked open‐ended questions like: What do you think of white rice? What do you think of brown rice? What are the differences between white rice and brown rice? Why do you think people eat white rice instead of brown rice on a regular basis? What do you think of different effects of eating brown rice on health? In addition, on the day of FGD, participants first completed a questionnaire about demographics including age (in years), sex (male/female), marital status (married, single and divorced), ethnicity, professional background, number of family members, total monthly income, and vegetarian or nonvegetarian status.

#### 2.3.2 Post‐taste FGD

The purpose of the post‐taste FGD was to recognize employees’ perception of brown rice after tasting and identify factors influencing substitution of white rice with brown rice. The post‐taste FGD guide covered the following major themes like: (1) perception about brown rice, (2) barriers to brown rice introduction, (3) factors to promote brown rice acceptance, and (4) side effects of brown rice. A moderator (PG, or DT, or AS) asked open‐ended questions like: What do you think of brown rice compared to white rice? What was your experience of eating brown rice? What do you think of brown rice as a substitute for white rice? We are planning to introduce Brown rice in the canteen. How can we make brown rice more preferable? What kind of brown rice recipes would you prefer on a regular basis?

The investigators using an iterative process discussed each interview shortly after it was completed and made suggestions for future interviews, with subsequent interviews probing more deeply into themes emerging in earlier interviews. A moderator (PG, DT, or AS) facilitated the 45‐60 minute FGDs (before and after tasting) in a private room in Nepali language using the semistructured interview guide. The FGDs were audio‐recorded in a tablet.

### 2.4 Rice preparation and tasting

We prepared five combinations of white and brown rice: 100% white rice (100W); 75% white rice and 25% brown rice (25B); 50% white rice and 50% brown rice (50B), 25% white rice and 75% brown rice (75B), and 100% brown rice (100B). A chef prepared the rice along with mixed vegetables, lentil soup, and spicy salad following a standard recipe (equal amount of water, oil, salt, condiments, and raw vegetables). On the day of tasting, the cook prepared the rice in a steamer after soaking for 30 min. Brown and white rice were prepared separately and mixed before serving. A total of 200 grams of cooked rice was served with seasonal vegetables, lentil soup, and spicy salad in each meal.

Each participant tasted the randomly selected rice combinations during the lunch hour on 5 different days with a gap of 3 days in between each tasting session. The participants were not informed about the combination of rice they tasted. For the rice tasting, participants were seated around a table. Each table was occupied by four participants. No other food and beverages were provided during tasting except water.

### 2.5 Rice rating

The participants used a hedonic rating scale to rate the rice for appearance, taste, aroma, texture, and overall impression. Although the reliability of hedonic rating scale was not assessed, it is deemed appropriate for wide spectrum of population and participant can answer meaningfully without the need of having past experiences (Peryam, & Pilgrim, 1957). The score ranged from 1 to 9, with 1 as “dislike extremely,” 5 as “indifferent,” and 9 as “like extremely.” The rating was done in two parts—first, participants consumed two tablespoons of rice only and then reported overall as well as individual rating of rice preparations; and second, the participant consumed the rice preparation served with lentil soup and vegetables and then rated the rice preparation in order to obtain their ratings on rice as it is usually served in Nepal.

### 2.6 Data analysis

We entered the data of demographics characteristics and hedonic rating for each rice combination in the Microsoft Excel and computed mean, standard deviation in STATA. PG transcribed the audio recordings verbatim into the Microsoft word and in Nepali language; and DT reviewed the transcript and compared them against the recording. We used inductive and deductive thematic coding to allow findings to emerge from frequent, dominant, or significant themes inherent in the raw data. Data were analyzed using the thematic framework method to identify the themes related to brown rice perception, and the facilitators and barriers to substituting brown rice for white rice. The investigators PG, DT, and AS read through the transcripts several times to familiarize themselves with the data. The text was then divided into meaningful units, such as phrases and quotes, and the meaningful units were then condensed, and the condensed meaningful units were abstracted and labeled with codes. Out of eight transcripts, two pretasting FGD (PG and AS) and two post‐tasting FGDs (DT and AS) were double coded independently; the intercoder reliability was 86% and 82%, respectively. As the agreement rate was high, the rest of the transcripts were single coded. The various codes were compared on the basis of differences and similarities and sorted into categories. The categories were further discussed by the investigators (PG and AS) for identification and formulation of themes and subthemes.

## 3 RESULTS

### 3.1 Characteristics of the study participants

Table 1 shows the characteristics of the study participants. The mean age was 33 years and 71 percent were female. More than half of participants were of the Newar ethnicity and nonvegetarian. About 63% of participants had Bachelor or Master's degree and 67% were married. The median monthly income was USD 543.5.

**TABLE 1:** Characteristics of adults in Nepal participating in a taste test of rice preparations in mean (*SD*) and proportion

Characteristics	Participants *n* = 42
Age (mean/*SD*), in years	32.7 (6.4)
Sex	
Male	12 (28.6)
Ethnicity	
Brahmin	8 (19.0)
Chettri	10 (23.8)
Newar	22 (52.3)
Magar	2 (4.7)
Education	
Masters	14 (32.3)
Bachelors	13 (30.9)
High school	6 (14.2)
Less than high school	9 (21.4)
Marital Status	
Married	28 (66.7)
Monthly Income in USD, median (IQR) (NPR 115 = 1$)	543.5 (608.7)
Number of Family Members (mean/ *SD*)	5.3 (2.1)
Diet Preference	
Vegetarian	5 (11.9)

### 3.2 Hedonic Scale rating after tasting rice preparations of various white to brown rice ratios

**FIGURE 1:**
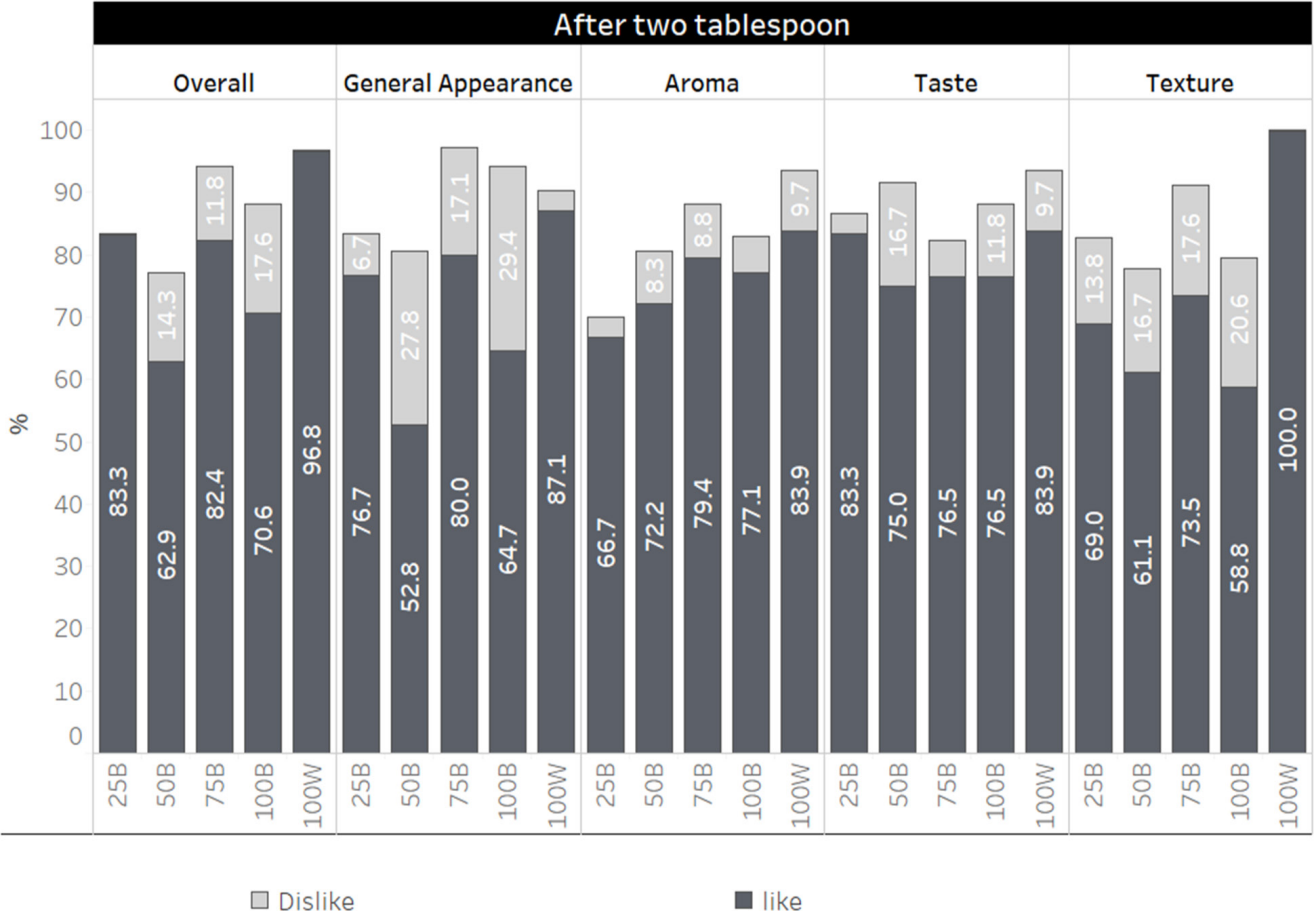
Hedonic scale rating after tasting two tablespoons of rice preparations of various white to brown rice ratios

Figure 1 presents a hedonic rating on aroma, general appearance, overall, taste, and texture after two tablespoons of various proportions of brown and white rice in combination. Overall, 97% liked the 100W preparation, 71% liked the 100B, and 83% liked the mixture of 25B. The 100W preparation received the highest score on aroma, general appearance, taste, and texture. About 83% of participants liked the taste and overall impression of 25B combination (Table 2).

**TABLE 2:** Emerging themes in before‐ and after‐taste FGDs

Pretasting	Post‐tasting
Brown rice perception	Brown rice perception
Unappealing appearance, bland taste, unpleasant smell, rough texture, food for diabetics, food for the low income	Unappealing appearance, bland taste, pleasant smell, rough texture.
Barriers to brown rice acceptance	Barriers to brown rice introduction
Poor perception of brown rice Discomfort while eating Lack of awareness about nutritional value Cooking method and time Tradition Household size	Price Unavailability, Lack of knowledge about health benefits Family member; children and elderly in the family Habituation to white rice
Facilitators to brown rice acceptance	Factors to promote brown rice acceptance
Availability of brown rice Awareness of health benefits Education of family members Diabetes in family Mixture of brown and white rice Habit formation	Tasting brown rice Awareness of brown rice and its health benefits Improved appearance, cooking method and serving style • Soaking prior to cooking • Serving hot and with varied side dishes, e.g., meat • Frying with soya sauce, turmeric Gradual replacement of white rice

**FIGURE 2:**
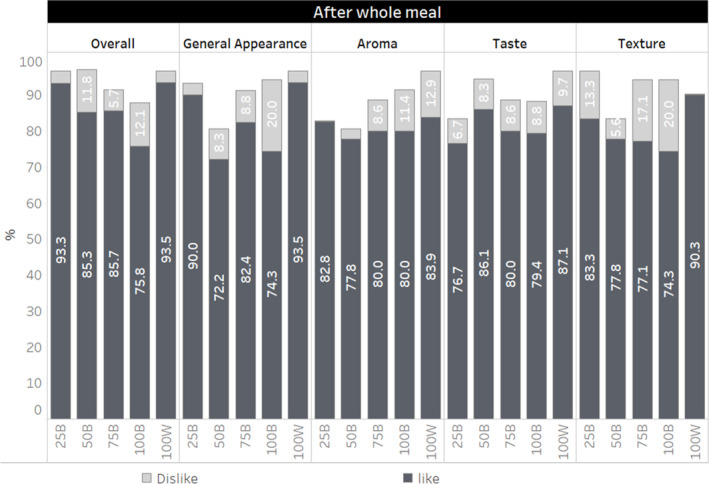
Hedonic scale rating after tasting rice preparations of various white to brown rice ratio in whole meal combinations

Figure 2 presents a hedonic rating on aroma, general appearance, overall, taste, and texture after whole meal consumption of various ratios of brown and white rice in combination. Overall, 94% of participants liked 100W, 76% liked 100B, and 93% liked mixture of white rice and brown rice in the ratio 25B after the whole meal consumption. The 100W preparation received the highest score on aroma, general appearance, taste, and texture. Ninety three percent liked the combination of 25B, which was similar to the 100W. More than 80% of participants reported to like the aroma and texture of 25B.

### 3.3 Perception about brown rice before and after tasting

#### 3.3.1 Before‐taste

a. Most participants thought that brown rice is unappealing because of its color, taste, smell, and rough texture compared to white rice. Most participants said that brown rice smells unpleasant. Some of the participants who had tasted brown rice previously found it to be tasteless.

I think, in comparison to white rice, brown rice is tasteless

It [brown rice] feels hard like it hasn't been cooked enough, just like stone lacking softness

b. *Food for people with diabetes or low income:* Most participants in the study perceived brown rice to be a diet for people with diabetes. One participant also mentioned it as a food for people of low income.

This rice is consumed by people who have sugar (diabetes)

In some places in society, it is thought to be food for the poor

#### 3.3.2 After‐taste

- After tasting, most participants thought that brown rice tasted good and some mentioned that it is possible to eat. Some found no difference in the taste between brown and white rice whereas few said they liked 100B. Regarding appearance, almost all participants found it unappealing compared to white rice but few mentioned it felt firm and hard in texture. All agreed 100B smelled better than they previously thought.

Brown rice isn't that distasteful as we thought initially.

Most importantly, I like the plain brown rice more than the mixture.

### 3.4 Facilitators to brown rice acceptance before and after tasting

#### 3.4.1 Before‐taste

a. *Availability of brown rice:* Participants mentioned that if brown rice were readily available, it would be easier to switch from white rice.

If it [brown rice] was available in the workplace canteen], we would have definitely eaten.

b. *Awareness on the health benefits of brown rice:* Participants knew that brown rice is good for health, few were able to mention its health benefits. Five of them mentioned it retains the outer layer, compared to white rice, therefore contains more vitamins and antioxidants. Few mentioned it is high in fiber content. One said brown rice provided satiety for a longer duration, and another said it helps in controlling obesity. Furthermore, two medical practitioners pointed out that brown rice takes longer time to digest, thereby regulating blood sugar level.

I think it controls obesity

I feel eating brown rice makes you feel hungry a little later.

If brown rice is good for health compared to white rice, then it is possible to eat.

c. *Educating family members:* The participants mentioned it was important to educate their family members on the health benefits of brown rice to encourage acceptance.

Even the family members are ready as they are aware of diabetes.

d. *Diabetes in the family*: Few participant mentioned that it would be easier to promote brown rice if somebody in the family has diabetes.

There is no one in my family who has gotten diabetes, in my family if anyone had diabetes, we would have bought and we would have known about brown rice.

In my family my mother has got diabetes and she eats “roti” in the evening, brown rice could replace that.

e. *Mixture of brown and white rice*: Some participants suggested that brown rice be mixed with white rice to begin with in order to promote acceptance.

It is better to mix little amount of brown rice with white rice, rather than not eating at all.

f. *Habit formation*: Although they were accustomed to the white rice, several participant believed that by gradually introducing brown rice into their diet they would become acclimated to it.

If we start consuming brown rice, definitely at some point we will get used to it.

#### 3.4.2 After‐taste

a. *Tasting:* After tasting the brown rice, participants were more willing to try brown rice.

Initially it [brown rice] tasted bland but later I felt it was possible to eat.

b. *Awareness of brown rice and its health benefit*: Participants stated that there is a lack of knowledge on brown rice. Its acceptability would be aided by increasing public understanding of its health benefits.

There is not much advertisement of brown rice in the market.

A public awareness campaign about the benefit and drawback of eating brown rice should be organized.

c. *Improved appearance, cooking and serving style:* After tasting, participants were keen on sharing cooking tips to enhance the taste and appearance of brown rice. They suggested cooking small amounts at a time and soaking rice prior to cooking made it softer, frying with soya sauce and/or turmeric would improve its appearance, and serving rice hot with a variety of side dishes including meat would enhance taste.

If the rice is cooked at once, in a larger amount it will lower the quality.

If brown rice is fried and served, then it may increase acceptability.

If we are making fried rice, then we can make it colorful so that the bad appearance of rice is masked by the effect of color.

d. *Gradual introduction of brown rice:* Participants emphasized brown rice would be better accepted if it was introduced gradually giving them time to get accustomed. One idea they mentioned was to mix it with white rice at first to improve appearances and reduce cost.

If we use the brown rice and white rice mixture it will be easier for a single or joint family.

If we start brown rice right away from tomorrow nobody will go to the canteen.

### 3.5 Barriers to introduction and acceptance of brown rice before and after tasting

#### 3.5.1 Before‐taste

a. *Poor perception of brown rice:* The poor perception of the appearance, taste, smell, and longer cooking time of brown rice were common barriers to acceptance among our study participants both before and after tasting.

If I have to choose rice on the basis of appearance, I wouldn’t choose brown rice.

The brown rice grains are larger and rough, which makes it difficult to swallow.

b. *Discomfort of eating brown rice*: Before tasting, few participants mentioned that brown rice causes discomfort such as constipation, difficulty in swallowing, and throat discomfort. One participant mentioned stomach discomfort after eating brown rice in the past, deterring him/her from accepting brown rice.

I don't think it does much harm, however, i think it causes constipation

I heard while eating brown rice it causes discomfort in the throat and also difficulty in swallowing.

c. *Lack of awareness of the nutritional value of brown rice:* Very few participants were able to identify the health benefits of brown rice. Few participants stated that brown rice has less carbohydrate.

I think brown rice has less carbohydrate, therefore is consumed by people with diabetes.

d. *Cooking method and time:* One participant who had eaten brown rice before stated that brown rice takes longer time to cook while another mentioned that it feels hard and sticky.

I have cooked brown rice once or twice, when it gets cold it feels firm and sticky.

it takes longer time to cook.

e. *Traditions of eating white rice:* Most said the long‐standing traditions of eating white rice made switching from white to brown rice difficult. The strong preference for white rice was tied to the habit of eating white rice since childhood. One participant commented that eating white rice has been passed from generation to generation and it has become a tradition.

Our culture and practices are such that we are used to eating white rice and brown rice is not practice.

f. *Household Size*: According to some participants, larger number of family member would discourage them from eating brown rice daily as it would be difficult to persuade all family members and also increases the household cost.

It may be easier to convince a single family, but it is difficult to convince every member of joint family.

It becomes expensive for everyday use in a joint family.

#### 3.5.2 After‐taste

a. *Price*: Almost all participants either did not know the price of brown rice or thought that it was more expensive than white rice.

I have bought white rice, but i haven't bought brown rice, I have no idea what is the cost of brown rice in the market.

I think it is comparatively more expensive than the white rice.

b. *Unavailability of brown rice:* Participants mentioned that the comparative unavailability of brown rice in the market discouraged participants from eating brown rice. Moreover, one participant said that the common milling process produces and supplies white rice to the market, making it abundantly available.

The main thing is—it is not available in the nearby store where we purchase white rice regularly.

These days the milling system removes it all and we import white rice from India.

c. *Lack of knowledge of health benefits:* One participant mentioned that the lack of knowledge of the health benefit of brown rice would discourage them from using brown rice.

Until and unless they understand the health benefits they are not going to eat it

d. *Family member*: Participants felt meals are a family affair and changing them needed everyone's approval. After tasting the brown rice, some of them mentioned the rough texture of brown rice would make it particularly difficult for young children and the elderly to eat it.

If we eat brown rice at home, we are not sure if younger children and older members will be able to eat or are going to like it.

It is difficult to convince everybody in the family to eat brown rice everyday.

e. *Habituation to white rice*: After tasting brown rice, participant said it was important to regularly eat brown rice to change their habit of eating white rice.

It is just that we are not habituated, once we are adapted to it we can eat.

## 4 DISCUSSION

This study aimed to assess perceptions, facilitators, and barriers to introducing brown rice in regular meal in place of white rice in Nepal. Before tasting the brown rice, the participants perceived it as poor in quality (taste, aroma, appearance, and texture) compared to white rice. However, after tasting, the participants expressed that the brown rice was better than they perceived before. From the hedonic scale rating, the taste, aroma, appearance, and texture of 100% white rice was scored the highest; however, mixtures of 75% white rice and 25% brown rice were rated higher on aroma, texture, appearance, and smell compared to 100% brown rice. The most commonly reported facilitators for switching from white to brown rice were easy availability, self and family awareness about the health benefits, knowledge about the brown rice cooking process and serving with side dishes, and introducing brown rice in combination with white rice and gradual increasing the brown rice proportion. The barriers to accepting brown rice were poor perception of its quality, the idea that brown rice is food for those with diabetes and the poor, a lack of awareness about the benefit of brown rice, higher price, unavailability, and traditional preference for white rice.

A person's rice preference is guided by sensory appeal of its color, taste, texture, and aroma (Sudha et al., 2013; Zhang et al., 2010). A high correlation between hedonic ratings on sensory attributes of brown rice by US consumers and trained panel have been established (Schutz & Oamrell, 1974). In our study, 100% white rice was the most preferred. Likewise, in Costa Rica, participants preferred beans to white rice in the ratio 1:1 more than that of the 1:1 beans to brown rice (Monge‐Rojas et al., 2014). However, brown rice in combination with 75% white rice was rated close to 100% white rice in terms of overall impression and aroma, suggesting that brown rice can be accepted by introducing it in combination with white rice.

Before tasting, our participants viewed the brownish color and rough texture of brown rice as an indication of poor quality, as previously reported (Kumar et al., 2011). The initial poor perception of brown rice changed after tasting brown rice, even among those tasting it for the first time. The post‐tasting focus group discussion revealed that despite initial hesitation, study participants were willing to switch to brown rice, especially after learning about its health benefits. The participants suggested adapting cooking methods to make brown rice appealing and palatable to consumers. The initial opinion of brown rice changed after tasting; the taste and smell improved suggesting that the gradual substitution of white rice was acceptable. Furthermore, the participants reported that wide availability of brown rice would increase the acceptance of brown rice. We found that availability and affordability (cost) also increased the acceptability of brown rice (Muhihi et al., 2012).

The lack of awareness about the nutritional properties of brown rice affected the preference for rice variety (Sudha et al., 2013; Zhang et al., 2010). Our study participants perceived brown rice as a food for diabetes patients. University hospital employees from Tanzania also associated brown rice as a therapeutic food for diabetic patients (Muhihi et al., 2012). Partial knowledge about nutritive properties of brown rice among hospital employees accustomed to prescribing or observing brown rice being prescribed to diabetes patients’ diet may have led to such perception. The outer layer of brown rice consists of vitamins, minerals, and fiber; more than half of it is lost during the milling process in producing white rice (Babu et al., 2009). A ½ cup of brown rice contains around 1–1.9g of dietary fiber while that of a cup of white rice consists of less than 1 g (Slavin & Green, 2007). In addition, brown rice is a good source of protein with balanced amino acids and higher quantity of unsaturated fatty acids, minerals, bioactive compound, and antioxidants as compared to white rice (Saleh et al., 2019).

Our study participants lacked knowledge about the nutritional benefits of brown rice. Increased brown rice consumption has been found to reduce the risk of type 2 diabetes, for managing body weight (Kazemzadeh et al., 2014) and improving lipid levels (Malik et al., 2019). Participants, particularly those with a medical background, knew the health benefits of eating brown rice. They were willing to replace white rice for brown rice after understanding its nutritional properties. Also, our study participants suggested that educating family members about the health benefits of brown rice will increase its acceptance. Likewise, in India, participants who learnt the nutritional benefits of brown rice through health and nutrition education were willing to switch to brown rice if it was inexpensive (Sudha et al., 2013).

Traditions and customs are known to determine food choices of people (Muhihi et al., 2012). Our study found that tradition and culture practices as an established barrier to brown rice consumption and indicated strong cultural preference and habituation to white rice. Not only in Nepal is white rice cherished as a staple food. Consumption of white rice has been central to Costa Rican tradition (Monge‐Rojas et al., 2014), sticky white rice is highly consumed in China (Zhang et al., 2010), and parboiled and raw white rice is traditional in Southern India (Kumar et al., 2011). Previously, people consumed hand‐pounded or brown rice in Nepal. With modernization and introduction of rice mills, polished white rice gained popularity. Now it has become the preferred choice, an inherent part of culture and tradition as in the rest of southeast Asia. Furthermore, with the advent of new milling technology, white rice is abundantly available in the market (Saleh et al., 2019).

Our study reported that unavailability and the higher cost of brown rice are the major barriers to introduction and sustaining brown rice consumption. Although the outer layer of brown rice is nutrient dense, it is also more susceptible to infestation by insects and becomes rancid quickly which limits prolonged storage in the market and thus its availability (Das et al., 2012). Another explanation for unavailability is due to low demand from consumers who are unaware about the health benefits of brown rice. A study from the Philippines reported lack of rice milling technology, local market, high price, poor quality, and lack of knowledge as constraints for the supply chain of brown rice (Das et al., 2012). A similar market analysis in Nepal is needed to understand factors that affect the supply chain of brown rice.

Household size and age of family members also influences the preference for brown rice. Our participants consistently reported that it is difficult to convince all family members to eat brown rice. In addition, we found that brown rice may be more accepted if there is someone with diabetes in the family. Studies from China and India both found that brown rice would be considered by older and people with health problem, and it would be more sustainable if approved by all family members (Kumar et al., 2011; Zhang et al., 2010). In Nepali context, women are entirely responsible for preparing meals throughout the day. In our study also, women were more concerned if the family members are also contended with the sudden dietary change as observed in Costa Rica (Monge‐Rojas et al., 2014).

Before tasting, some participants mentioned that they believed that brown rice caused throat and stomach discomfort and constipation. However, no side effects were reported after tasting. The throat discomfort may be due to the rougher texture of brown rice compared to white rice. However, the belief that it causes constipation might be an outlier as studies have found that brown rice based diet enhance bowel movement in those with constipation (Jung et al., 2020). Abdominal pain, diarrhea, gassiness, thirst, borborygmic, and headache were reported in another study after consuming brown rice meal in higher proportion (Adebamowo et al., 2017). The participants reported that, compared to white rice, they felt satiated for a longer duration after eating brown rice. The high fiber content in brown rice slows the rate of digestion of carbohydrate and its absorptions (Chandalia et al., 2000; Panlasigui & Thompson, 2006) and slows the passage of food from stomach to small intestine during the process of digestion (Pletsch & Hamaker, 2018) lowering the postprandial blood glucose, improving insulin sensitivity (Weickert & Pfeiffer, 2008), and stimulating satiety (Pletsch & Hamaker, 2018).

This is the first study from Nepal to report on brown rice perceptions and acceptance. The mixed‐method approach incorporating qualitative and quantitative approaches is the strength of our study. In addition, tasting brown rice provided grounds for perception and acceptability. A limitation of this study is that it was conducted among hospital employees limiting generalization of the result. We also did not assess the reliability of hedonic rating scale in our population.

These findings suggest that the promotion of brown rice would best occur in a stepwise process. Acceptance of brown rice challenges long‐established food habits. Brown rice can be introduced in the worksite cafeteria by gradually substituting with a mixture of brown and white rice. It is also important to pay attention to the taste by developing recipes of brown rice that are palatable and acceptable for daily consumption. Promoting knowledge of brown rice's nutritive properties and health benefits can help improve awareness and uptake of brown rice among Nepali consumers.

## CONFLICT OF INTEREST

The authors have no conflict of interest to declare.

## AUTHOR CONTRIBUTION

**Pratiksha Gyawali:** Conceptualization (lead); Data curation (lead); Formal analysis (lead); Funding acquisition (lead); Investigation (lead); Methodology (lead); Project administration (lead); Resources (lead); Software (lead); Supervision (lead); Validation (lead); Visualization (lead); Writing – original draft (lead); Writing – review & editing (lead). **Dipesh Tamrakar:** Conceptualization (equal); Data curation (equal); Formal analysis (equal); Investigation (equal); Methodology (equal); Project administration (equal); Resources (equal); Software (equal); Supervision (equal); Validation (equal); Visualization (equal); Writing – original draft (equal). **Abha Shrestha:** Conceptualization (supporting); Data curation (supporting); Formal analysis (supporting); Investigation (supporting); Methodology (supporting); Project administration (supporting); Supervision (equal); Writing – review & editing (equal). **Himal Shrestha:** Data curation (equal); Formal analysis (equal); Project administration (supporting); Validation (equal); Visualization (equal); Writing – review & editing (equal). **Sanju Karmacharya:** Conceptualization (supporting); Data curation (equal); Formal analysis (supporting); Investigation (equal); Methodology (equal); Project administration (equal); Resources (equal). **Sanju Bhattarai:** Writing – review & editing (supporting). **Niroj Bhandari:** Data curation (supporting); Formal analysis (supporting). **Vasanti Malik:** Writing – review & editing (supporting). **Josiemer Mattei:** Writing – review & editing (supporting). **Donna Spiegelman:** Writing – review & editing (supporting). **Archana Shrestha:** Conceptualization (equal); Data curation (equal); Formal analysis (equal); Funding acquisition (equal); Investigation (equal); Methodology (equal); Project administration (equal); Resources (equal); Software (equal); Supervision (equal); Validation (equal); Visualization (equal); Writing – original draft (equal); Writing – review & editing (equal).

## Data Availability

The data that support the findings of this study are available from the corresponding author upon reasonable request.
